# Site-specific association between distal aortic pulse wave velocity and peripheral arterial stenosis severity: a prospective cardiovascular magnetic resonance study

**DOI:** 10.1186/s12968-014-0095-8

**Published:** 2015-01-20

**Authors:** Harrie C M van den Bosch, Jos J M Westenberg, Wikke Setz-Pels, John Wondergem, Ron Wolterbeek, Lucien E M Duijm, Joep A W Teijink, Albert de Roos

**Affiliations:** Department of Radiology, Catharina Hospital, Michelangelolaan 2, 5623EJ, Eindhoven, The Netherlands; Department of Radiology, Leiden University Medical Center, Leiden, The Netherlands; Department of Medical Statistics, Leiden University Medical Center, Leiden, The Netherlands; Department of Radiology, Canisius-Wilhelmina Hospital, Nijmegen, The Netherlands; Department of Vascular Surgery, Catharina Hospital, Eindhoven, The Netherlands

**Keywords:** Cardiovascular magnetic resonance, Atherosclerosis, Peripheral arterial occlusive disease, Pulse wave velocity, Carotid vessel wall

## Abstract

**Background:**

Vascular disease expression in one location may not be representative for disease severity in other vascular territories, however, strong correlation between disease expression and severity within the same vascular segment may be expected. Therefore, we hypothesized that aortic stiffening is more strongly associated with disease expression in a vascular territory directly linked to that aortic segment rather than in a more remote segment. We prospectively compared the association between aortic wall stiffness, expressed by pulse wave velocity (PWV), sampled in the distal aorta, with the severity of peripheral arterial occlusive disease (PAOD) as compared to atherosclerotic markers sampled in remote vascular territories such as PWV in the proximal aorta and the normalized wall index (NWI), representing the vessel wall thickness, of the left common carotid artery.

**Methods:**

Forty-two patients (23 men; mean age 64±10 years) underwent velocity-encoded cardiovascular magnetic resonance (CMR) in the proximal and distal aorta, whole-body contrast-enhanced MR angiography (CE-MRA) and carotid vessel wall imaging with black-blood CMR in the work-up for PAOD. Strength of associations between aortic stiffness, carotid NWI and peripheral vascular stenosis grade were assessed and evaluated with multiple linear regression.

**Results:**

Stenosis severity correlated well with PWV in the distal aorta (Pearson r_P_=0.64, p<0.001, Spearman r_S_=0.65, p<0.001) but to a lesser extent with PWV in the proximal aorta (r_P_=0.48, p=0.002, r_S_=0.22, p=0.18). Carotid NWI was not associated with peripheral stenosis severity (r_P_=0.17, p=0.28, r_S_=0.14, p=0.37) nor with PWV in the proximal aorta (r_P_=0.22, p=0.17) nor in the distal aorta (r_P_=0.21, p=0.18). Correlation between stenosis severity and distal aortic PWV remained statistically significant after correction for age and gender.

**Conclusions:**

Distal aortic wall stiffness is more directly related to peripheral arterial stenosis severity than markers from more remote vascular territories such as proximal aortic wall stiffness or carotid arterial wall thickness. Site-specific evaluation of vascular disease may be required for full vascular risk estimation.

## Background

It is well known that the expression of vascular disease in one location may not be representative for the severity of disease in other vascular territories. From an observational cardiovascular magnetic resonance (CMR) study in 394 subjects, Barbier et al. reported that unrecognized myocardial infarction was not associated with manifestation of atherosclerosis depicted on whole-body MR angiography, nor with increased intima-media thickness (IMT) sampled in the carotid artery [1].

However, strong correlation has been reported between vascular disease expression and vascular wall changes within the same vascular segment. Increased wall thickness and wall stiffening in the carotid artery have been associated with the presence of atherosclerotic plaque in patients with hypertension and elderly patients [2]. Additionally, a stronger association between arterial vessel wall thickness and wall stiffness has been demonstrated when these markers were sampled regionally within the same vascular territory of either the aorta or the carotid artery, rather than across vascular territories [3]. Atherosclerosis involves both arterial wall thickening due to fatty degeneration (i.e., atherosis) and arterial wall stiffening due to media degeneration (i.e., sclerosis) [4,5]. Atherosclerosis is therefore not limited to luminal narrowing and structural changes in the arterial wall, but is also strongly associated with arterial wall stiffening [6]. The pulse wave velocity (PWV) has been acknowledged as an important indicator for increased aortic stiffness with prognostic value for cardiovascular events [7,8]. With velocity-encoded CMR, the PWV can be accurately assessed with high reproducibility, regionally in the aorta [9].

We hypothesized that aortic stiffening is more strongly associated with the expression of vascular disease in the vascular territory at risk directly linked to that aortic segment rather than in a more remote aortic segment or in other vascular territories. Accordingly, the purpose of this study was to prospectively compare the association between aortic wall stiffness, expressed by pulse wave velocity, sampled in the distal aorta with the severity of peripheral arterial occlusive disease as compared to atherosclerotic markers sampled in remote vascular territories such as PWV in the proximal aorta and normalized wall index [10] describing the vessel wall thickness of the left common carotid artery.

## Methods

### Patients

In our study, 42 consecutive patients (23 men; mean age 64±10 years) were included who were clinically referred for CE-MRA evaluation and were either suspected for PAOD due to clinical symptoms or already known to be suffering from PAOD and had to undergo follow-up evaluation. In all patients, a single comprehensive CMR examination was performed consisting of a moving-table CE-MRA of the run-off vessels, carotid vessel wall imaging and assessment of the aortic pulse wave velocity. In all patients, the glomerular filtration rate (GFR) was >60 mL/min/1.73 m^2^. No adverse reactions or complications occurred during or after MRA. Institutional Review Board approval and written informed consent was obtained from all patients.

Of note, 16 patients of the present study have been described previously in a study comparing different MRA techniques of the run-off vessels [11].

### CMR protocol

CMR was performed using a 3T CMR system (Achieva X-series, release 2.1; Philips Healthcare, Best, The Netherlands). Whole-body CE-MRA was performed and has been partially described before [11]. In short, first standardized 3-station single-injection CE-MRA was performed, including the abdominal aorta, iliac arteries and run-off vessels. The contrast protocol consisted of a biphasic contrast injection using an CMR-compatible injector (Spectris MR injector; Medrad, Indianola, PA). In total, 0.1 mmol/kg body weight gadoterate meglumine (Gd-DOTA, Guerbet, Paris, France) was administered. The first half of the contrast bolus was administered at 1.2 mL/s and the remaining half at 0.5 mL/s. Contrast injection was followed by 15 mL saline flush at 0.6 mL/s. Timing of the contrast arrival, required to start the acquisition of the CE-MRA, was determined by means of automatic bolus timing (BolusTrak; Philips Healthcare, Best, The Netherlands). For signal transmission and reception a quadrature body coil was used in all three stations. Imaging parameters of the 3D fast gradient-echo (FFE) CE-MRA were as follows: for the pelvic arteries repetition time (TR) 3.6 ms, echo time (TE) 1.25 ms, flip angle 20°, field-of-view (FOV) 410 mm, acquired voxel size 1.25×1.84×3.70 mm^3^, reconstructed voxel size 0.73×0.73×1.85 mm^3^; for the upper-leg arteries TR/TE 3.6/1.26, flip angle 20°, FOV 410 mm, acquired voxel size 1.30×1.75×3.00 mm^3^, reconstructed voxel size 0.73×0.73×1.50 mm^3^;for the lower-leg arteries TR/TE 4.7/1.60, flip angle 30°, FOV 410 mm, acquired voxel size 0.80×0.90×1.40 mm^3^, reconstructed voxel size 0.71×0.71×0.70 mm^3^. The first station was acquired with reversed linear k-space filling, the second and third station were acquired with centric k-space filling. Table speed was set at 180 mm/s between all imaged stations. The fourth station consisting of the thoracic aorta and its supra-aortic braches, including the carotid arteries, was acquired after a second contrast injection (0.1 mmol/kg body weight Gd-DOTA). Timing of contrast arrival was determined by means of automatic bolus timing. For signal transmission and reception a quadrature body coil was used. Imaging parameters were as follows: TR/TE 5.5/1.80, flip angle 30°, FOV 410 mm, acquired voxel size 0.96×0.97×2.00 mm^3^, reconstructed voxel size 0.64×0.64×1.00 mm^3^. Data were acquired with centric k-space filling.

**Figure 1:**
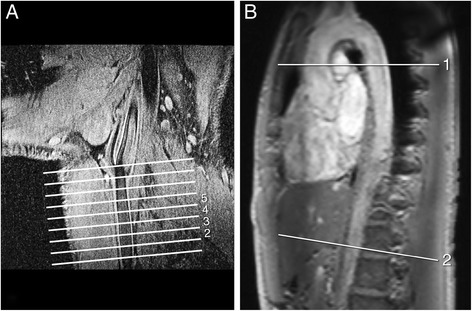
**Carotid vessel wall thickness and aortic pulse wave velocity. A**: Multi-slice 2D Black-Blood CMR of the common carotid artery for assessment of vessel wall thickness. Eight cross-sectional slices of the left common carotid artery were acquired, with the last slice adjacent to the bifurcation, however only slice 2 to 5 were included in the analysis. **B**: Proximal and distal aortic Pulse Wave Velocity (PWV) was assessed by two one-directional velocity-encoded CMR acquisitions in through-plane direction, planned perpendicular to the aorta at the level of the pulmonary trunk and at the abdominal aorta, respectively.

Preceding to the whole-body CE-MRA procedure, the vessel wall of the left common carotid artery was examined in all patients using multi-slice two-dimensional black blood imaging. On oblique sagittal and oblique coronal survey scans axial slices were planned perpendicular to the course of the common carotid artery (Figure 1A). Starting from the carotid flow divider, eight contiguous slices of 2 mm thick were acquired in caudal direction. To maximize contrast between the carotid vessel wall, the lumen blood pool and the surrounding tissue, a 2D dual-inversion-recovery (black-blood) gradient echo technique with spectral selective fat suppression was used. VCG-triggering was used for gated data acquisition at end-diastole. For signal reception, a 2-element Flex-M surface coil was positioned around the neck of the patient. Images were acquired at each RR interval with the following typical parameters: TR/TE 12/3.5, flip angle 45°, FOV 140 mm, two signal averages, acquired voxel size 0.46×0.46×2 mm^3^.

The pulse wave velocity, defined as the wave propagation speed, was assessed regionally in the aorta, between the ascending and descending thoracic aorta (i.e., the proximal aorta), and between the thoracic descending and distal abdominal aorta (i.e., the distal aorta) (Figure 1B), using a 6-element cardiac coil. Two one-directional through-plane velocity-encoded CMR acquisitions were performed to assess the flow waveform propagation through the aorta. First, a double-oblique sagittal survey of the aorta was obtained (1C) and used for planning of the velocity-encoded acquisitions. The first velocity-encoded acquisition was planned perpendicular to the ascending aorta at the level of the pulmonary trunk, and transecting both the ascending and thoracic descending aorta. The second acquisition was planned at the most distal aortic location still present in the sagittal survey. Velocity-encoded CMR was performed using a retrospectively VCG-gated gradient-echo sequence with velocity-encoding in through-plane direction: TR/TE 4.9/3.0, flip angle 10°, FOV 320 mm, one signal average, acquired voxel size 2.50×2.50×8.00 mm^3^. The maximum number of phases was reconstructed in order to obtain high temporal resolution (the true temporal resolution was 2×TR =9.8 ms). At the proximal level, a velocity sensitivity V_enc_ of 150 cm/s was used and at the distal level, V_enc_ =100 cm/s. Free breathing was allowed during the acquisition.

### Image analysis

**Figure 2:**
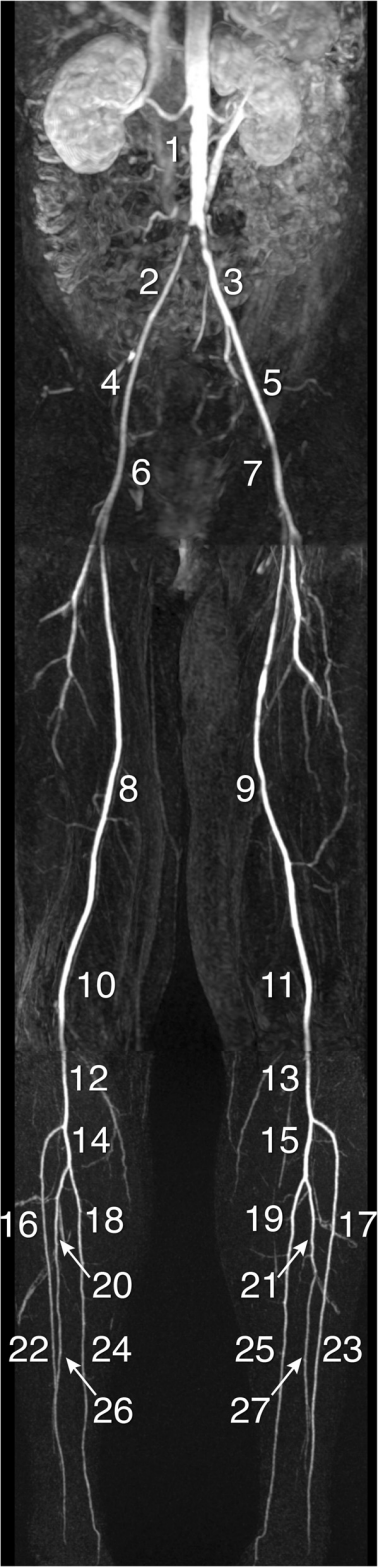
**Segments of arterial tree from infrarenal aorta to peripheral arteries.** Contrast-enhanced MRA of the infra-renal aorta down to the lower leg arteries in a patient with a subtotal stenosis in the right common iliac artery. For each patient, mean and maximal stenosis class were obtained by dividing the arterial tree into 27 segments: the infrarenal aorta (1), common iliac arteries (2 +3), external iliac arteries (4 +5), common femoral arteries (6 +7), superficial femoral arteries (8 +9), popliteal arteries in the thigh station (10 +11), popliteal arteries in the calf station (12 +13), tibiofibular trunk (14 +15), and the proximal (16-21) and distal (22-27) halves of the anterior and posterior tibial arteries and peroneal arteries.

MR angiographic images were reviewed at random and in consensus by two MR radiologists (HvdB and JW; 15 and 17 years of experience with CE-MRA, respectively). CE-MRA images were analyzed on a remote workstation. The arterial tree from infrarenal aorta down to the peripheral arteries was divided into 27 segments (Figure 2): the infrarenal aorta, common iliac arteries, external iliac arteries, common femoral arteries, superficial femoral arteries, popliteal arteries in the thigh station, popliteal arteries in the calf station, tibiofibular trunk, and the proximal and distal halves of the anterior and posterior tibial arteries and peroneal arteries. The severity of each stenosis was visually graded according to a five point scale: class 1 (0%-stenosis), 2 (1-50%), 3 (51-75%), 4 (76-99%) and 5 (100%). The highest stenosis class per segment was determined, and next, the highest stenosis class over all available segments (maximal 27), presenting one value per patient (Max SC), was determined. Also, the mean stenosis class (Mean SC), averaged over all available segments was calculated. While both parameters relate to the severity of PAOD, Max SC relates to the stenosis severity and Mean SC relates to distribution of stenoses in peripheral arterial tree. Stenosis classification per patient was performed blinded from carotid vessel wall and aortic PWV analysis.

Cross-sectional carotid vessel wall area (VWA) and total vessel area were obtained using the previously validated in-house developed software package VesselMass software (Leiden University Medical Center, Leiden, the Netherlands [12]. Inner and outer lumen contours were manually defined. Analysis was performed in four slices out of eight. The mean cross-sectional carotid VWA and total vessel area were averaged over the four included slices, and subsequently the normalized wall index (NWI) [10] was calculated as follows: NWI=VWA/total vessel area.

The aortic PWV was obtained from systolic wave propagation analysis based on the transit-time method which has been validated before [9]. The aortic path length (∆x), describing the distance between sampling sites in the ascending and the thoracic descending aorta for proximal aortic PWV (PWV_proximal_) and between the thoracic and abdominal descending aorta for distal aortic PWV (PWV_distal_), respectively, was manually determined using MASS software (Leiden University Medical Center, Leiden, The Netherlands), by placing a poly-line along the centerline of the aorta in the sagittal survey images. Each aortic path length measurement was performed twice and averaged. Wave propagation was evaluated from maximal velocity-time curves that were obtained at each sampling site by using FLOW software (Leiden University Medical Center, Leiden, the Netherlands) with automated contour detection for image segmentation. The transit-time defining the wave propagation was assessed from the onset of each wave front, automatically calculated from the intersection of the horizontal line modeling the diastolic flow (averaged over the final 250 ms of the velocity-time curve) and the upslope of the systolic wave which was modeled by a straight line, using linear regression of all values between 20% and 80% of the range of velocity values that are part of the upslope.

### Statistical analysis

Kolmogorov-Smirnov tests were performed to test normality of data distribution. Continuous variables are expressed as mean ± standard deviation (SD). Associations between atherosclerotic disease severity (Mean SC and Max SC) and atherosclerotic markers PWV_proximal_, PWV_distal_ and carotid NWI were explored. Furthermore, association between NWI and PWV was examined. Correlation between Mean SC and Max SC and PWV_proximal_, PWV_distal_ and carotid NWI was examined by calculating Pearson (for continuous variables) or Spearman (in case of association with an ordinal variable) correlation coefficients, where appropriate. Multiple linear regression analyses were performed with Mean SC as dependent variable and age, gender and respectively alternating proximal and distal aortic PWV and carotid NWI as predictors. Additionally, interaction between predictors was analyzed. A p-value of <0.05 is considered statistically significant. Statistical analysis was performed using IBM SPSS software version 20 (Armonk, NY, USA).

## Results

**Figure 3:**
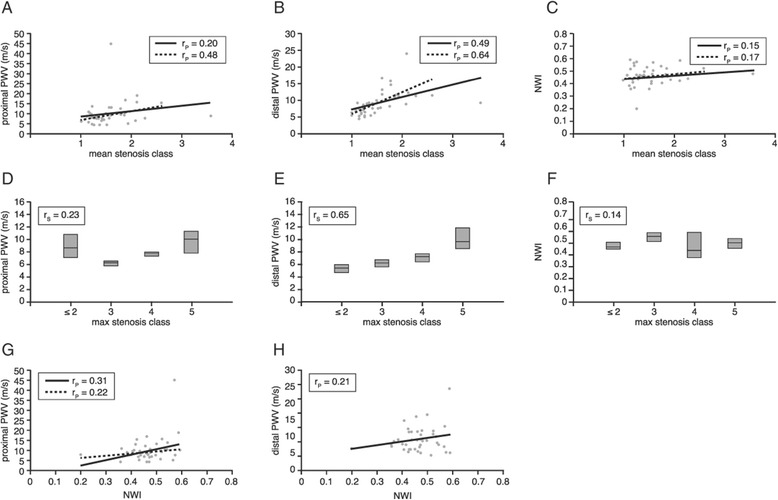
**Association stenosis severity, PWV, and carotid wall thickness (A-H).** Associations between stenosis severity (expressed in mean and max stenosis class), the proximal and distal aortic pulse wave velocity and normalized wall index sampled in the left common carotid artery. Solid line represents correlation between parameters for the full dataset, dashed line represents correlation for dataset with two outliers removed.

Patient characteristics are presented in Table 1. Thirty-four patients (81%) presented with intermittent claudication (Fontaine classification 2), of which twenty-three patients (55%) with pain-free claudication walking ≥200 m and eleven patients (26%) with pain-free claudication walking <200 m. Five patient (12%) presented with rest pain (Fontaine classification 3) and three patients (7%) with necrosis (Fontaine classification 4). Mean ankle-brachial index (ABI) in rest was 0.75±0.21. CE-MRA was successful in all patients. No imaging artefacts or venous contamination affected the image analysis and image quality was sufficient for stenosis scoring in all segments. Mean SC per patient ranged between 1 (no stenosis) and 3.6, with a mean value ± standard deviation of 1.6 ±0.5. Max SC ranged between 1 and 5, with mean ± SD 4.4 ±1.0 (median 5, interquartile range from 4 to 5). Carotid NWI ranged from 0.20 to 0.59, with mean ± SD of 0.46 ±0.07. PWV ranged from 4.5 m/s to 45.3 m/s in the proximal aorta (mean ± SD 10.1±6.5 m/s) and from 4.4 m/s to 24.4 m/s in the distal aorta (mean ± SD 9.3 ±3.8 m/s). All continuous data were normally distributed according to Kolmogorov-Smirnov tests (all p>0.05). In Figure 3, scatter plots and box plots are presented to illustrate data distribution and associations between markers. From the distribution of the data in these figures it is obvious that one outlier for Mean SC (Figures 3A to 3C: data point with Mean SC =3.6) and one outlier for PWV in the proximal aorta (Figures 3A, 3D and 3G: data point with PWV =45.3 m/s) might contribute substantially to the correlations. For the patient with Mean SC of 3.6, PWV_proximal_ was 8.8 m/s, PWV_distal_ 9.2 m/s and carotid NWI 0.48. For the patient with PWV_proximal_ 45.3 m/s, the Mean SC was 1.6, PWV_distal_ 13.2 m/s and carotid NWI 0.57. Analysis was performed on the full dataset as well as on the dataset with these two outliers removed. For the data with outliers removed, mean values ± SD changed for Mean SC 1.5 ±0.4 and for PWV_proximal_ 9.3 ±3.5 m/s.

**Table 1:** **Patient characteristics**

Gender (male/female)	23/19
Age (years)	64 ± 10
Hypertension (yes/no)	28/14
Fontaine Class (stages I/IIa/IIb/III/IV)	0/23/11/5/3
ABI in rest	0.75 ± 0.21
Mean SC	1.6 ± 0.5
Max SC (median (interquartile range))	5 (4−5)
Carotid NWI	0.46 ± 0.07
PWV_proximal_ (m/s)	10.1 ± 6.5
PWV_distal_ (m/s)	9.3 ± 3.8

*ABI*: ankle-brachial index; *SC*: stenosis class; *NWI*: normalized wall index; *PWV*: pulse wave velocity.

In two patients, significant stenoses were found in the carotid arteries: in one patient, a class 3 stenosis was depicted in the right internal carotid artery and in the other patient, a full occlusion was present in the right internal carotid artery while the left internal carotid artery presented with a class 3 stenosis. All other patients were free from stenoses in the carotid arteries. In 28 patients, no stenosis was present in the distal aorta; 11 patients were graded with a class 2 stenosis in the abdominal aorta, 1 patient with class 3 stenosis, 1 patient with class 4 stenosis and one patient with an occlusion. Spearman correlation coefficients indicated no significant correlations between stenosis severity in the distal aorta and proximal aortic PWV (r_S_=−0.09, p=0.59) and distal aortic PWV (r_S_=−0.21, p=0.21), respectively. Low correlation was found between stenosis severity in the distal aorta and the carotid NWI (r_S_=0.38, p=0.02).

In two patients, no stenoses were depicted on any of the CE-MRA images. Correlations between Mean SC in the complete run-off and proximal and distal aortic PWV and carotid VWA/BSA were evaluated by Pearson correlation coefficients, while correlations between Max SC, proximal and distal aortic PWV and carotid NWI were evaluated by Spearman correlation coefficients, respectively. The results are presented in Table 2, both for all data point as well as for the data with two outliers removed.

**Table 2:** **Pearson and Spearman correlation coefficients between mean and maximal stenosis severity, proximal and distal aortic PWV and cross-sectional carotid NWI, both for the total dataset as well as for one outlier removed**

	**All patients included**			**Two outliers removed**		
	**Mean SC r**_**P**_	**Max SC r**_**S**_	**NWI r**_**P**_	**Mean SC r**_**P**_	**Max SC r**_**S**_	**NWI r**_**P**_
**PWV**_**proximal**_	0.20 (p=0.22, N=42)	0.23 (p=0.14, N=42)	0.31 (p=0.048, N=42)	0.48 (p=0.002, N=40)	0.22 (p=0.18, N=41)	0.22 (p=0.17, N=41)
**PWV**_**distal**_	0.49 (p=0.001, N=42)	0.65 (p<0.001, N=42)	0.21 (p=0.18, N=42)	0.64 (p<0.001, N=41)	0.65 (p<0.001, N=42)	0.21 (p=0.18, N=42)
**NWI**	0.15 (p=0.34, N=42)	0.14 (p=0.37, N=42)		0.17 (p=0.28, N=41)	0.14 (p=0.37, N=42)	

*PWV*: pulse wave velocity, *NWI*: normalized wall index, *SC*: stenosis class, r_P_: Pearson correlation coefficient, r_S_: Spearman correlation coefficient, *N*: number of samples.

None to moderate association between PWV in the proximal aorta and stenosis class (r_P_=0.46 for Mean SC and r_P_=0.22 for Max SC) was found. Associations between distal aortic PWV and stenosis class are good. However, associations are absent between carotid NWI and stenosis class as well as between carotid NWI and proximal and distal aortic PWV.

In Table 3, the results of multiple linear regression between Mean SC and proximal and distal aortic PWV, carotid NWI, age and gender are presented. No significant interaction was found between these predictors (all p>0.05). These tests show that proximal and distal aortic PWV and carotid NWI are all statistically significantly associated with age, but none of these predictors is associated with gender. However, for these patients, only distal aortic PWV remained significantly associated with Mean SC (with β=0.46, p=0.003), when corrected for age (β=0.30, p=0.04) and gender (β=−0.06, p=0.61).

**Table 3:** **Multiple linear regression analysis with mean stenosis class as dependent variable and age, gender and respectively alternating cross-sectional carotid normalized wall index, proximal and distal aortic pulse wave velocity as predictors**

	**NWI**				**PWV**_**proximal**_				**PWV**_**distal**_		
	**B (SE)**	**β**	**p**		**B (SE)**	**β**	**p**		**B (SE)**	**β**	**p**
**Constant**	0.13 (0.39)		0.75	**Constant**	0.31 (0.33)		0.36	**Constant**	0.40 (0.29)		0.18
**Age**	0.02 (0.005)	0.50	0.001	**Age**	0.02 (0.01)	0.41	0.01	**Age**	0.01 (0.005)	0.30	0.04
**Gender**	−0.15 (0.11)	−0.21	0.17	**Gender**	−0.11 (0.10)	−0.15	0.28	**Gender**	−0.04 (0.09)	−0.06	0.61
**NWI**	0.59 (0.76)	0.12	0.44	**PWV**_**proximal**_	0.03 (0.02)	0.27	0.08	**PWV**_**distal**_	0.05 (0.01)	0.46	0.003

*NWI*: normalized wall index, *PWV*: pulse wave velocity, *SE*: standard error.

## Discussion

This study prospectively evaluated the association between aortic wall stiffness, expressed by PWV, sampled in the distal aorta and the severity of PAOD as compared to atherosclerotic markers sampled in remote vascular territories such as the PWV in the proximal aorta and vessel wall thickness of the left common carotid artery. The main findings of our study are: 1) In patients with PAOD, the PWV in the distal aorta is well associated with peripheral arterial stenosis severity. 2) Correlation between peripheral arterial stenosis severity with markers sampled in remote vascular territories such as PWV in the proximal aorta and carotid VWA NWI, are weak to absent, only moderate at best, indicating site-specific association between aortic wall stiffness and peripheral arterial disease severity. 3) Only the correlation between stenosis severity and distal aortic PWV remains statistically significant after correction for age and gender.

PAOD is an important clinical manifestation of atherosclerosis, for which 3T CE-MRA is a reliable diagnostic tool, widely used to detect and grade stenosis severity [11]. However, CE-MRA is not without risks, especially in patients with impaired renal function [13]. The initial test in clinical routine for diagnosing patients with clinical symptoms of PAOD is assessment of the ankle-brachial index (ABI) [14]. A low ABI is a strong indicator of the presence of PAOD [15] but a normal ABI, however, does not rule out risk due to the false negative rates [16]. Furthermore, Wikström et al. reported that ABI<0.9 may underestimate the prevalence of PAOD when assessed with CE-MRA [17]. Therefore, the evaluation of other markers associated with the severity of PAOD is warranted. Atherosclerosis is a systemic disease which affects the arterial wall both by thickening and stiffening. Structural changes in the wall usually occur over a larger part of the arterial tree rather than confined to a localized arterial segment (i.e., diffuse preintrusive wall thickening in contrast to focal intrusive thickening [2]). Maroules et al. recently demonstrated a strong association between wall thickness sampled in the abdominal aorta and the occurrence of cardiovascular events in a large population-based study [18]. Additionally, arterial wall stiffening has been described as an independent predictor of cardiovascular events in patients with hypertension, diabetes and end-stage renal disease [19,20] as well as in the aging population [21]. Moreover, increased carotid arterial wall thickness is associated with carotid wall stiffening and with the presence of atherosclerotic plaque in the carotid artery and the aorta in patients with hypertension and elderly patients [22].

However, it is well known that the expression of vascular disease in one location may not be representative for the severity of disease in other vascular territories and it is conceivable that disease severity is stronger associated with atherosclerotic markers sampled within the same vascular territory than when sampled in a more remote vascular territory. Barbier et al. demonstrated that unrecognized myocardial infarction is not necessarily associated with atherosclerotic disease severity, depicted from whole-body MR angiography nor with increased intima-media thickness (IMT) sampled in the carotid artery [1]. Furthermore, in a recent publication, Turkbey et al. found no independent association between imaging biomarkers of atherosclerosis such as carotid IMT and distensibility sampled in the ascending aorta [23]. This finding was confirmed by our results, as the association between carotid NWI and the PWV in the proximal aorta was not statistically significant. Furthermore, in a study by Brandts et al. it was shown both in healthy volunteers as well as in patients with hypertension that the aortic PWV correlated more strongly with the aortic VWA than when compared to the carotid VWA [24]. Kröner et al. [3] confirmed these findings in healthy volunteers and added stronger correlations between arterial vessel wall thickness and stiffness when sampled within the same vascular territory of either the aorta or the carotid artery, rather than across vascular territories. These reports underline the suggestion of a strong site-specific coupling between morphologic and functional degenerative changes of the arterial wall and the possible effect on future cardiovascular events.

In our study, 42 consecutive patients referred for CE-MRA for the evaluation of severity of PAOD were included. In these patients, the prevalence of significant stenoses in the distal aorta was low (i.e., 7%), and therefore, no association with PWV in this part of the aorta could be demonstrated. However, the stenosis severity detected with CE-MRA in the peripheral arteries correlated well with the PWV sampled in the distal aorta, but to a lesser extent with PWV in the proximal aorta, and no association was found with carotid NWI. Taniwaki et al. [25] showed that in patients with type 2 diabetes mellitus, the presence of PAOD symptoms was more closely associated with increased femoral arterial wall stiffness compared to increased femoral arterial wall thickness, suggesting that stiffening has a significant impact on clinical symptoms in patients with peripheral vascular disease. In our study, aortic wall stiffness is represented by the PWV, which is defined as the systolic wave front velocity propagating through the aorta. The assessment of PWV by CMR has shown good agreement with invasive pressure measurements, the gold standard for defining PWV [9,26]. Still, data on reproducibility of this assessment is scarce. Grotenhuis et al. [9] evaluated the reproducibility by repeating acquisitions on the same day and reported a coefficient of variation of 9% for PWV assessment in the total aorta by CMR. In a study by Suever et al. [27], reproducibility of CMR-assessed PWV was compared to applanation tonometry by performing repeated studies on volunteers on the same day. However, no data has been published for repeated acquisitions on separate days, therefore information on the physiological variation of this marker is missing, although CMR-assessed PWV is widely used in clinical research. To our knowledge, this is the first clinical study to evaluate the association between peripheral arterial stenosis severity and regional aortic wall stiffness and carotid vessel wall thickness, all assessed from one comprehensive CMR examination. The results from our study and from literature underline a potentially important role for the site-specific assessment of arterial wall stiffness by PWV regionally in the aorta as this may be required for full estimation of vascular risk in patients with atherosclerosis. The strong site-specific association between distal aortic wall stiffening and the degree of stenosis in the peripheral arteries suggests that sampling within the same vascular territory may be preferred over sampling across vascular territories.

We acknowledge certain limitations of our study. First, it involves a cross-sectional design in a relatively small prospectively included patient population in which the association between atherosclerotic markers and stenosis severity was evaluated. Association, however, does not prove causality. Population-based long-term follow-up studies are required to prove whether distal aortic stiffening indeed is a precursor to PAOD and to elucidate the predictive value of increased wall stiffness in the distal aorta with respect to further development of flow-limiting stenoses in the peripheral arteries. Carotid arterial vessel wall sampling was limited to the left common carotid artery. Prevalence of stenoses in the carotid circulation in this patient population was very low. Adding VWA sampling of the right carotid artery would be possible but this was not performed, however, as it requires additional scan time. Also, no comparison with ultrasound-assessed IMT was performed, additionally to VWA sampling with Black-Blood CMR, but from literature it is known that CMR shows good agreement with ultrasound when sampling IMT [28]. The higher spatial resolution on ultrasound potentially may improve the association between IMT and PAOD stenosis severity, however, CMR has the advantage of providing increased vessel coverage with information on the complete circumference over the length of a vessel, which permits assessment of localized abnormalities without assuming vessel uniformity.

## Conclusions

Peripheral arterial stenosis severity is well correlated with aortic PWV sampled in the distal aorta but correlation with markers sampled in remote vascular territories such as PWV in the proximal aorta and carotid arterial wall thickness, is only moderate at best. The association between aortic wall stiffening and stenosis severity in a vascular territory directly linked to this aortic segment suggests that site-specific evaluation of vascular disease may be required for full vascular risk assessment and therefore, regional aortic PWV assessment by CMR may be considered in the clinical workup of PAOD.

## Abbreviations

ABI: Ankle-brachial index
BSA: Body surface area
CE-MRA: Contrast-enhanced MR angiography
FFE: Fast gradient-echo
FOV: Field-of-view
GFR: Glomerular filtration rate
IMT: Intima-media thickness
Max Sc: Highest stenosis class per patient
Mean Sc: Mean stenosis class per patient
NWI: Normalized wall index
PAOD: Peripheral arterial occlusive disease
PWV: Pulse wave velocity
TE: Echo time
TR: Repetition time
VWA: Vessel wall area
